# Municipal Meat Inspection

**Published:** 1900-05

**Authors:** 


### THE JOURNAL

OF

### COMPARATIVE MEDICINE AND VETERINARY ARCHIVES.

VOL. XXI. *MAY, 1900.* No. 5.

[Written especially for the JOURNAL.]

### MUNICIPAL MEAT INSPECTION.

THE commercial spirit may be credited with the establishment, in this country, of Federal meat-inspection. A better milk-supply has been the result of the dealer's desire to hold his trade. It will be commercialism rather than health that will compel municipal inspection of meat, and the compelling agent will be the physician. This proposition is therefore submitted, that the physician is the most important factor at present in the development of municipal meat-inspection.

Like all other branches of the science of medicine, that of dietetics has had a gradual development, and it is still in process of evolution.

There was the time when, barring milk-punch or the broths, the physician wisely left dietetic suggestions to the old nurse, with the mere direction that the patient be kept on a low diet or "fed up." Now the physician writes his diet lists for diabetes, rheumatism, the various dyspepsias, gout, uric acid, Bright's disease, etc. Accordingly he makes his suggestions, now for a beef diet, again for a milk diet, at another time for a diet consisting entirely of vegetables. He goes further. He distinguishes between meats of long fibre and short fibre, proscribing the one, prescribing the other. So he goes over the vegetable foods, making selected lists for the needs of different classes of patients.

It was not at all out of place, a number of years ago, for Dr. Sydney Ringer to introduce into his therapeutics a department of dietetics in which were found simple but excellent recipes for the dietary of the sick.

"Must the physician be a nurse?" used to be many times asked. It has come to be answered that the physician who understands the art of nursing will, other things being equal, secure beyond his confrères the public confidence. "Must the physician be a cook?" I suppose was asked when Ringer appended his dietary to his therapeutics. While he may not need to be capable of engaging as chef to the Waldorf-Astoria, experience has demonstrated the wisdom of the physician being, at least, enough of a cook to direct, when necessary, in the preparation of articles of food for his patient.

There was a time when the quality of the milk was left, by the physician, entirely to the cow. Where he found that the quality of the milk was modified by the mercenary spirit of the dealer, the physician secured the passage of laws intended to prevent this. When the veterinarian had proved the existence in the cow of certain diseases, like tuberculosis, and had demonstrated the possibility of contagion through the milk, the physician made a demand for the inspection of dairy herds. He has learned now that milk is a remarkable carrier of disease through germs introduced from without, and, knowing that the greatest possible care cannot altogether prevent this, he has begun to recommend a Pasteurized milk. And it is in response to the physician's demands, based upon facts demonstrated by scientific investigation, that milk dealers are compelled to-day to offer a milk made pure by Pasteurization.

It is not too much to expect that the same history will be repeated in regard to meat. The time must come when the physician shall be as well posted on all the diseases which affect the food value of all food-producing animals as he now is on the diseases which are generally traced directly or indirectly to the milk-supply. The time is almost here when the physician will be as well acquainted with the purposes and methods of meat-inspection as he now is with milk-inspection. And then he will begin to demand inspection of meat, and he will express that demand through the laws of the Board of Health. And when that time comes we shall see that the physician will keep himself informed as to the houses from which inspected meat comes, and urge that meat, and that only, upon his patients. Then, the pocketbook of the butcher being touched in a legitimate way, the dealer himself will court inspection as the abattoirs now ask for Federal inspection in connection with interstate trade.

The veterinarian has done his work and done it so well that in a number of instances States and cities have responded to that work in the passage of laws requiring inspection of meats. But in many places political jobbery has stepped in, and men unfitted for the work have been appointed. It is simply another case of laws being vitiated in their operation by their premature birth—that is, by being enacted ahead of an enlightened sentiment. And that enlightened sentiment in regard to meat-inspection must be secured through the agency and co-operation of the physician. Hence a repetition of the proposition with which this began: the physician is the most necessary factor in the further development of municipal meat-inspection.

It is useless to attempt to refute the charge that in advocating inspection of meats by trained veterinarians a "job" is wanted. Too often have government positions been developed on that principle, and the world is too busy to stop and minutely investigate individual character and motives. It makes its judgments wholesale.

What, then, is needed? Just this: That the physician shall be trained in the necessity and methods of meat-inspection, and so be placed in position to intelligently advocate it. The graduated physician must be trained through the articles in medical journals and through papers and addresses at society meetings. This was urged a year ago by an honored member of the profession; but you cannot compel a man to read medical journals, nor can you compel him to attend society meetings. So let us direct our work upon the coming physician. Through the few who possess the influence rather than through the many, let us urge the establishing in medical schools of lectureships on milk-inspection and meat-inspection. Let them be at first optional courses. And let us, as the men fitted by training and practice, especially those who are inspectors, hold ourselves ready to offer even gratuitous service on this line. Here is the opportunity to disprove the charge above referred to. There is work in such a lectureship, but an interest in the welfare of the community should lead us to be satisfied in the pioneer days of such a movement to put forth labor the appreciation of which may not be in dollars and cents.

It is a pleasure to recall the Harvard Medical School, and to point to its endowed chair of comparative pathology, whose incumbent, Dr. Theobald Smith, for years did worthy service in the Bureau of Animal Industry. This is a story more in the direction advocated by this paper. There may be other medical schools that have made such addition to their studies, but it has not been the fortune of the writer to see their curricula.

Let every member of the profession, every qualified meatinspector, go forth with this sentiment: milk-inspection and meat-inspection taught in every medical school in the land. I would not have it taught in the same minute manner that it should be taught in veterinary schools, but *have it taught.* It might be, for a while, only an arm of the chair of hygiene, but *have it taught.* It might be but a leg of the chair of dietetics, but *have it taught.* Surely the incumbent of either the chair of hygiene or the chair of dietetics would be glad to have this course supplemented by a few lectures and demonstrations by a trained inspector—in some way, in any way—on milk-inspection and meat-inspection in every medical school in the land. Then, as surely as day follows night, meat-inspection will be called for by an intelligent popular demand, fostered by the sentiment of the medical profession.

